# Distribution characteristics of MTHFR C677T gene polymorphism in Han nationality and women of childbearing age in 8 regions of Shaanxi province and a comparative analysis with other regions in China

**DOI:** 10.3389/fgene.2025.1693729

**Published:** 2025-11-13

**Authors:** Ting Chen, Fei Song, Jinyao Sun, Jing Yi, Wenbing Ma

**Affiliations:** 1 Pharmacy Intravenous Admixture Services, Pharmaceutical Department, The First Affiliated Hospital of Xi’an Jiaotong University, Xi’an, Shaanxi, China; 2 Department of Pharmacology, The First Affiliated Hospital of Xi’an Jiaotong University, Xi’an, Shaanxi, China; 3 Department of Genetic Laboratory, Baoji Maternal and Child Health Hospital, Baoji, Shaanxi, China

**Keywords:** Shaanxi province, women of childbearing age, MTHFR C677T, genetic polymorphism, geographical distribution

## Abstract

**Objective:**

To analyze the distribution characteristics of MTHFR C677T gene polymorphism in Shaanxi Province, thereby providing a genetic basis for folic acid supplementation strategies and the reduction of adverse pregnancy outcomes among reproductive-aged women in this region.

**Methods:**

A total of 8,779 Han women of childbearing age were recruited from the First Affiliated Hospital of Xi’an Jiaotong University and Baoji Maternal and Child Health Hospital for MTHFR C677T genotyping. The genotype and allele distribution patterns across eight regions in Shaanxi were systematically compared with domestic and international populations.

**Results:**

In the Shaanxi Province, the genotype frequencies of MTHFR C677T polymorphism ranked as follows: CT (48.49%), TT (29.22%), and CC (22.29%). Allelic frequencies were 46.54% (C) and 53.46% (T). Significant regional variations in MTHFR C677T genotype frequencies (P < 0.05) were observed among the eight prefectures of Shaanxi. Notably, the Xi’an region exhibited distinct genotype and allele distribution patterns compared to most reported Chinese populations (P < 0.05).

**Conclusion:**

There are significant interregional differences in the distribution of MTHFR C677T genotypes and alleles across Shaanxi Province. The genetic polymorphism pattern of MTHFR C677T genotypes and allele frequencies in Han women of reproductive age in Xi’an is significantly distinct from that in many other regions.

## 1 Introduction

Folic acid (FA), a crucial B-complex vitamin, serves as an essential cofactor in biological transmethylation reactions and participates in diverse physiological and pathological processes. Folate deficiency adversely affects female reproductive health through multiple mechanisms: increased uracil misincorporation into DNA, compromised nucleic acid integrity, delayed DNA replication, and elevated chromosomal fragility. Severe FA insufficiency during preconception and pregnancy periods may disrupt oocyte maturation, impair folliculogenesis, reduce endometrial receptivity, and ultimately compromise embryo implantation and fetal development (Lyazzat et al., 2024).

The methylenetetrahydrofolate reductase (MTHFR) gene, spanning 2.2 kb on chromosome 1p36.3, harbors the clinically significant rs1801133 (677C>T) polymorphism. A systematic review and meta-analysis showed that the total T allele frequency of MTHFR C677T rs1801133 in Chinese people is 36.9% (Yang et al., 2017), and 78.4% of Chinese people have homozygous or heterozygous mutations (Yang et al., 2017), which exceeds that in many other countries. The enzyme activity of individuals with homozygous TT mutations is approximately 30% of that of individuals with the wild-type (CC) genotype, while individuals with the heterozygous genotype (CT) have approximately 65% of the wild-type enzyme activity (Ulrich et al., 2000). Subsequently, the mutant genotype reduces the ability to convert 5,10-methylenetetrahydrofolate (5,10-MTHF) into 5-methyltetrahydrofolic acid (5-MTHF) (the main circulating form of folic acid), thus decreasing the utilization rate of folic acid (Mazokopakis and Papadomanolaki, 2020). Some studies have shown that the MTHFR C677T gene is a major genetic factor leading to adverse pregnancy outcomes (Kos et al., 2020; Du et al., 2019). In addition, research has found that the intake of FA from supplements can reduce the risk of spontaneous abortion and pregnancy complications (Mao et al., 2020; Serapinas et al., 2017). Currently, there are relatively few relevant research reports on a comprehensive analysis of the distribution of MTHFR C677T gene polymorphism among Han women of childbearing age in various regions of Shaanxi Province. This study aims to detect the MTHFR C677T gene in Han women of childbearing age in 8 regions of Shaanxi Province, explore the distribution characteristics of MTHFR C677T gene polymorphism in Shaanxi Province, and provide a genetic basis for folic acid supplementation among women of childbearing age in this region and for reducing adverse pregnancies.

## 2 Materials and methods

### 2.1 General information

The study recruited 8,779 Han Chinese women of childbearing age who underwent preconception (prenatal) check-ups at the First Affiliated Hospital of Xi’an Jiaotong University (September 2020 to April 2023) and Baoji Maternal and Child Health Hospital (January 2022 to April 2023). The mean age of participants was 30.24 ± 4.07 years. All subjects were of Han ethnicity native to the Shaanxi region and had no self-reported family history of genetic disorders.

### 2.2 Data source

- Except for the MTHFR gene polymorphism data of Xi’an, Xianyang, Tongchuan, Ankang, Shangluo, Hanzhong, Yulin, and Baoji, the data for other regions were retrieved from PubMed, Web of Science, China National Knowledge Infrastructure (CNKI), and Wanfang Science and Technology Journal Database. The English search terms were “pregnant woman”, “MTHFR”, and “Gene”, while the Chinese search terms included “childbearing age” (referring to women of childbearing age in this context), “MTHFR”, and “gene polymorphism”, etc. Each search term was connected with “and” (for English searches) or “和” (for Chinese searches) and combined as needed.

### 2.3 DNA extraction and genotyping

Venous blood (2 mL) was collected from patients into EDTA anticoagulant tubes. The blood samples were inverted and mixed several times. A 100 μL aliquot was transferred into 400 μL of sample extraction solution (CQ-ENH type, Jinan Guangyin Medical Technology Co., Ltd.), followed by vigorous inversion and shaking of the centrifuge tube 10 times to ensure thorough mixing. The mixture was then allowed to stand for 10 min on a tube rack to ensure complete erythrocyte lysis, resulting in a translucent red liquid. After standing, the supernatant was removed by centrifugation, completing leukocyte extraction. A 1.5 μL aliquot of the leukocyte suspension was added to a digoxin staining solution, thoroughly mixed, and briefly centrifuged. The tube was tightly sealed and loaded into a fluorescence detector (Fascan 48S multi-channel fluorescence quantitative analyzer, Xi’an Tianlong Technology Co., Ltd.). Fluorescence signal values were automatically analyzed using fluorescence *in situ* hybridization (FISH) and chromosomal karyotype analysis systems. Fluorescence curve graphs were generated to determine MTHFR C677T (677C>T) genotypes, with positive quality controls included.

### 2.4 Statistical analyses

The study population was tested for Hardy-Weinberg equilibrium. Statistical analyses were performed using SPSS 16.0 software. Quantitative data were expressed as mean ± standard deviation (x̄±s), while categorical data were presented as percentages. Intergroup comparisons were conducted using the chi-square (χ^2^) test. A P-value <0.05 was considered statistically significant.

## 3 Results

### 3.1 Hardy-Weinberg equilibrium analysis

The Hardy-Weinberg equilibrium test was performed on the MTHFR C677T gene locus in Han Chinese women of childbearing age from eight regions of Shaanxi Province. All results conformed to Hardy-Weinberg equilibrium (*P* > 0.05), demonstrating regional group representativeness of the childbearing-age populations in these areas (Table 1).

**TABLE 1:** MTHFR C677T genotype and allele frequency distribution among Han women of childbearing age in 8 regions of Shaanxi Province.

Region	MTHFR C677T genotype			Hardy-Weinberg				MTHFR C677T allele			
	CC	CT	TT	χ2	P	χ2	P	C	T	χ2	P
Xi’an	495	1,300	917	0.831	0.362	110.206	*P* < 0.01	2,290	3,134	107.616	*P* < 0.01
Xianyang	254	600	332	0.831	0.362			1,108	1,264		
Tongchuan	42	101	62	0.005	0.941			185	225		
Ankang	135	209	90	0.302	0.582			479	389		
Shangluo	193	339	195	3.302	0.069			725	729		
Hanzhong	97	173	62	0.964	0.326			367	297		
Yulin	166	305	144	0.029	0.865			637	593		
Baoji	575	1,230	763	3.496	0.062			2,380	2,756		
Total	1957 (22.29%)	4,257 (48.49%)	2,565 (29.22%)					8,171 (46.54%)	9,387 (53.46%)		

### 3.2 Comparison of MTHFR C677T genotype and allele distribution in 8 regions of Shaanxi Province

Among 8,779 tested samples, the MTHFR C677T genotypes were distributed in descending order: CT (48.49%), TT (29.22%), and CC (22.29%). The allele frequencies for C and T were 46.57% and 53.46%, respectively (Table 1). Significant regional variations in MTHFR C677T genotype frequencies (χ^2^ test) and allele distributions were observed across the eight regions of Shaanxi Province (*P* < 0.01).

### 3.3 Comparison of MTHFR C677T genotype and allele distribution among childbearing-age women in Xi’an, Shaanxi province, and other Chinese regions

**FIGURE 1:**
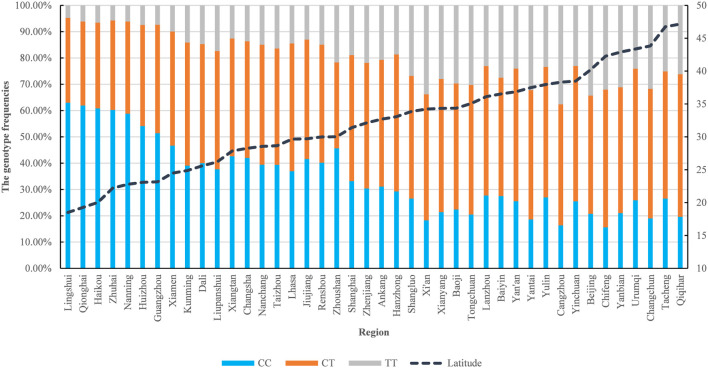
Distribution of C677T polymorphisms (CC, CT, TT) of MTHFR C677T gene in populations in various regions.

To investigate regional variations in MTHFR C677T genotype and allele frequencies between Han Chinese women of childbearing age in Xi’an, Shaanxi, and counterparts across China, comparative analyses were performed. Significant differences (*P* < 0.05) were observed in genotype/allele distributions between Xi’an and the following regions: Xianyang, Ankang, Shangluo, Hanzhong, Yulin, Baoji, Yan’an (Shaanxi); Wuhan, Zhoushan, Baiyin, Zhuhai, Qionghai, Zhenjiang, Kunming, Yanbian, Jiujiang, Qiqihar, Changsha, Lingshui County, Renshou County, Yinchuan, Taizhou, Guangzhou, Huizhou, Nanchang, Xiangtan, Liupanshui, Nanning, Lanzhou, Haikou, Beijing, Tacheng (Xinjiang), Shanghai, Dali (Yunnan), Xiamen, Urumqi, and Lhasa (Tibet). No statistically significant differences (*P* > 0.05) were detected compared to Tongchuan, Cangzhou, Yantai, Changchun, and Chifeng (Inner Mongolia). Notably, the TT homozygous genotype exhibited a distinct north-to-south, with higher frequencies observed in northern regions (Figure 1).

### 3.4 Allele frequency characteristics of MTHFR C677T in childbearing-age women from Xi’an, Shaanxi, and other Chinese regions

**FIGURE 2:**
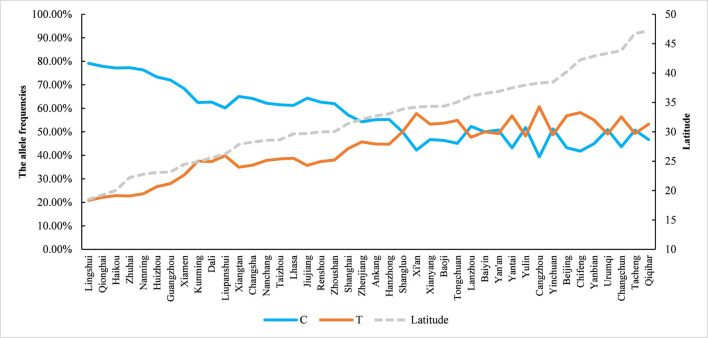
Distribution of C677T locus alleles (C, T) of MTHFR C677T gene according to latitude.

The allele frequencies of MTHFR C677T (C and T) in Han Chinese women of childbearing age from Xi’an, Shaanxi, significantly differed (*P* < 0.05) from those in the following regions: Xianyang, Ankang, Shangluo, Hanzhong, Yulin, Baoji, Yan’an (Shaanxi); Wuhan, Zhoushan, Baiyin, Zhuhai, Qionghai, Zhenjiang, Kunming, Yanbian, Jiujiang, Qiqihar, Changsha, Lingshui County, Renshou County, Yinchuan, Taizhou, Guangzhou, Huizhou, Nanchang, Xiangtan, Liupanshui, Nanning, Lanzhou, Haikou, Tacheng (Xinjiang), Shanghai, Dali (Yunnan), Xiamen, Urumqi, and Lhasa (Tibet). No statistically significant differences (*P* > 0.05) were observed compared to Tongchuan, Beijing, Cangzhou, Yantai, Changchun, and Chifeng (Inner Mongolia) (Table 2). Notably, the T allele frequency exhibited a negative latitudinal cline, decreasing progressively with lower geographic latitudes (Figure 2).

**TABLE 2:** Comparison of MTHFR C677T genotypes and allele distribution frequencies.

Region	例数(n)	Frequency of genotype distribution [n (%)]					Frequency of allele distribution [n (%)]			
		CC	CT	TT	χ2	*P*	C	T	χ2	*P*
Xi’an	2,712	495 (18.25)	1,300 (47.94)	917 (33.81)			2,290 (42.22)	3,134 (57.78)		
Xianyang	1,186	254 (21.42)	600 (50.59)	332 (27.99)	14.214	0.01	1,108 (46.71)	1,264 (53.29)	13.542	0
Tongchuan	205	42 (20.49)	101 (49.27)	62 (30.24)	1.311	0.519	185 (45.12)	225 (54.88)	1.314	0.252
Ankang	434	135 (31.11)	209 (48.16)	90 (20.74)	50.854	0	479 (55.18)	389 (44.82)	51.04	0
Shangluo	727	193 (26.55)	339 (46.63)	195 (26.82)	28.588	0	725 (49.86)	729 (50.14)	27.204	0
Hanzhong	332	97 (29.22)	173 (52.11)	62 (18.67)	40.425	0	367 (55.27)	297 (44.73)	40.969	0
Yulin	615	166 (26.99)	305 (49.59)	144 (23.41)	36.555	0	637 (51.79)	593 (48.21)	37.26	0
Baoji	2,568	575 (22.39)	1,230 (47.90)	763 (29.71)	18.121	0	2,380 (46.34)	2,756 (53.66)	18.152	0
Yan’an (Shi et al., 2020)	12,557	3,208 (25.55)	6,331 (50.42)	3,018 (24.03)	134.444	0	12,747 (50.76)	12,367 (49.24)	130.06	0
Zhoushan (Ye et al., 2022)	692	316 (45.66)	226 (32.66)	150 (21.68)	228.495	0	858 (61.99)	526 (38.01)	173.488	0
Baiyin (Zhang et al., 2022)	331	91 (27.49)	149 (45.02)	91 (27.49)	17.165	0	331 (50.00)	331 (50.00)	14.565	0
Zhuhai (Yin et al., 2016)	2,138	1,288 (60.24)	728 (34.05)	122 (5.71)	1,069.371	0	3,304 (77.27)	972 (22.73)	1,203.183	0
Cangzhou (Di et al., 2019)	606	99 (16.34)	279 (46.04)	228 (37.62)	3.474	0.176	477 (39.36)	735 (60.64)	3.341	0.068
Qionghai (Yan et al., 2013)	1,221	756 (61.92)	390 (31.94)	75 (6.14)	810.359	0	1902 (77.89)	540 (22.11)	860.592	0
Zhenjiang (Yang et al., 2012)	2,885	877 (30.40)	1,378 (47.76)	630 (21.84)	156.677	0	3,132 (54.28)	2,638 (45.72)	162.84	0
Kunming (Wan et al., 2013)	297	116 (39.06)	139 (46.80)	42 (14.14)	89.643	0	371 (62.46)	223 (37.54)	88.9	0
Yantai (Yan et al., 2014)	2,670	497 (18.61)	1,313 (49.18)	860 (32.21)	1.569	0.456	2,307 (43.20)	3,033 (56.80)	1.062	0.303
Yanbian (Yu et al., 2015)	2,620	551 (21.03)	1,253 (47.82)	816 (31.15)	8.165	0.017	2,355 (44.94)	2,885 (55.06)	8.038	0.005
Jiujiang (Fang et al., 2015)	1839	765 (41.60)	835 (45.41)	239 (13.00)	404.193	0	2,365 (64.30)	1,313 (35.70)	427.699	0
Qiqihar (Li et al., 2016)	367	72 (19.62)	199 (54.22)	96 (26.16)	8.71	0.013	343 (46.73)	391 (53.27)	5.374	0.02
Changsha (Wu et al., 2016)	1,586	666 (41.99)	704 (44.39)	216 (13.62)	366.305	0	2036 (64.19)	1,136 (35.81)	386.341	0
Lingshui (Huang et al., 2013)	1,275	803 (62.98)	412 (32.31)	60 (4.71)	882.082	0	2018 (79.14)	532 (20.86)	951.771	0
Renshou (Pan et al., 2017)	2,286	919 (40.20)	1,025 (44.84)	342 (14.96)	388.791	0	2,863 (62.62)	1709 (37.38)	413.391	0
Changchun (Tian et al., 2017)	3,405	648 (19.03)	1,676 (49.22)	1,081 (31.75)	2.975	0.226	2,972 (43.64)	3,838 (56.36)	2.491	0.115
Yinchuan (Yang et al., 2015)	443	113 (25.51)	228 (51.47)	102 (23.02)	25.105	0	454 (51.24)	432 (48.67)	25.223	0
Taizhou (Yang et al., 2021)	861	339 (39.37)	381 (44.25)	141 (16.38)	193.878	0	1,059 (61.50)	663 (38.50)	195.077	0
Guangzhou (Fu et al., 2019)	1,124	578 (51.42)	463 (41.19)	83 (7.38)	533.365	0	1,619 (72.02)	629 (27.98)	564.744	0
Huizhou (Zhong et al., 2022)	765	414 (54.12)	294 (38.43)	57 (7.45)	453.371	0	1,122 (73.33)	408 (26.67)	462.262	0
Nanchang (Xu et al., 2020)	2,213	872 (39.40)	1,010 (45.64)	331 (14.96)	368.762	0	2,754 (62.22)	1,672 (37.78)	390.318	0
Xiangtan (Wang et al., 2014)	1701	725 (42.62)	762 (44.80)	214 (12.58)	410.634	0	2,212 (65.02)	1,190 (34.98)	434.938	0
Liupanshui (Wang et al., 2017)	950	358 (37.68)	427 (44.95)	165 (17.37)	179.772	0	1,143 (60.16)	757 (39.84)	181.82	0
Nanning (Zhou et al., 2023)	4,157	2,445 (58.82)	1,457 (35.05)	255 (6.13)	1,435.797	0	6,347 (76.34)	1967 (23.66)	1,637.146	0
Lanzhou (Jia et al., 2021)	2018	559 (27.70)	993 (49.21)	466 (23.09)	92.221	0	2,111 (52.30)	1925 (47.70)	94.592	0
Haikou (Mo et al., 2018)	2,852	1735 (60.83)	930 (32.61)	187 (6.56)	1,230.853	0	4,400 (77.14)	1,304 (22.86)	1,413.932	0
Beijing (Li et al., 2019)	4,395	912 (20.75)	1976 (44.96)	1,507 (34.29)	8.62	0.013	3,800 (43.23)	4,990 (56.77)	1.401	0.237
Tacheng (Liu et al., 2021)	418	111 (26.56)	202 (48.33)	105 (25.12)	21.289	0	424 (50.72)	412 (49.28)	21.3	0
Chifeng (Wang et al., 2021)	647	101 (15.61)	339 (52.40)	207 (31.99)	4.701	0.095	541 (41.81)	753 (58.19)	0.073	0.788
Shanghai (Wang et al., 2021)	2,651	880 (33.20)	1,270 (47.91)	501 (18.90)	229.528	0	3,030 (57.15)	2,272 (42.85)	239.024	0
Dali (Yu et al., 2020)	3,203	1,281 (39.99)	1,451 (45.30)	471 (14.70)	461.833	0	4,013 (62.64)	2,393 (37.36)	492.224	0
Xiamen (Lin et al., 2020)	994	464 (46.68)	431 (43.36)	99 (9.96)	381.388	0	1,359 (68.36)	629 (31.64)	397.729	0
Urumqi (Hao et al., 2016)	216	56 (25.93)	108 (50.00)	52 (24.07)	12.171	0.002	220 (50.93)	212 (49.07)	12.384	0
Lhasa (Hao et al., 2016)	760	281 (36.97)	369 (48.55)	110 (14.47)	168.2	0	931 (61.25)	589 (38.75)	172.893	0

## 4 Discussion

Birth defects represent a persistent global medical challenge. It is widely recognized that their etiology stems from the complex and interactive effects of genetic, environmental, and nutritional factors. Among these, impaired maternal folate metabolism, leading to folic acid deficiency, is one of the most common factors with a genetic basis. Folic acid is an indispensable micronutrient for crucial physiological and biochemical processes during pregnancy, including DNA synthesis, protein metabolism, and embryonic development. Consequently, disturbances in the folate metabolic pathway can lead to adverse pregnancy outcomes, potentially jeopardizing the health of both the mother and the infant. MTHFR C677T is a pivotal enzyme in the folate metabolic pathway, catalyzing the irreversible conversion of 5,10-methylenetetrahydrofolate to 5-methyltetrahydrofolate, the primary circulatory form of folate essential for homocysteine remethylation (Yang et al., 2018). The MTHFR C677T polymorphism (rs1801133) is a common and clinically significant missense mutation involving a cytosine (C) to thymine (T) substitution at nucleotide position 677 in exon 4 of the MTHFR gene. This single nucleotide polymorphism (SNP) results in an alanine-to-valine amino acid substitution (Ala222Val) in the protein product. This structural change is known to impair the thermostability of the MTHFR enzyme, leading to its accelerated degradation. Consequently, this polymorphism significantly reduces the enzyme’s physiological activity, thereby disrupting normal folate metabolism and leading to folate metabolic disorders (Scime et al., 2020). Based on this C-to-T substitution, individuals can be classified into three distinct genotypes: wild-type homozygous (CC), heterozygous (CT), and mutant homozygous (TT) (Yang et al., 2017). In clinical practice, the MTHFR C677T genotype is used to detect impaired folate metabolism, specifically: the CC genotype indicates high enzymatic activity and normal folate conversion capacity; the CT genotype indicates intermediate enzymatic activity and folate conversion capacity; and the TT genotype indicates low enzymatic activity and folate conversion capacity. This impaired folate utilization due to the C677T mutation is strongly linked to a spectrum of adverse pregnancy outcomes (APOs), including hypertensive disorders of pregnancy (HDP), gestational diabetes mellitus (GDM), recurrent miscarriage, and fetal abnormalities such as neural tube defects (Yang et al., 2020; Huang et al., 2023). Therefore, investigating the role of the MTHFR C677T polymorphism in the pathogenesis of these conditions is of paramount importance. Screening women of reproductive age for this polymorphism holds significant value for tailoring individualized folic acid supplementation strategies, thereby providing a robust scientific rationale for the prevention and management of APOs.

This study analyzed the MTHFR C677T gene polymorphism in Han women of childbearing age in eight regions of Shaanxi. The results showed that the frequencies of MTHFR C677T C and T alleles in Han women of childbearing age in Shaanxi Province were 46.54% and 53.46% respectively; in addition, we also studied the distribution frequency of MTHFR C677T genotypes in 8,779 clinical samples, CT, TT, CC accounted for 48.49%, 29.22%, and 22.29% of the total number of samples respectively. There were significant differences in MTHFR C677T genotype frequencies and allelic differences among the 8 regions in Shaanxi Province, which provides critical evidence for optimizing local public health guidelines. First, we recommend incorporating MTHFR C677T genotyping into routine pre-pregnancy health screenings for women in Shaanxi, especially in regions with higher TT frequencies (e.g.,“xi’an and Tongchuan”). This targeted screening can help identify high-risk groups (TT carriers) early and avoid missed opportunities for intervention. Second, local public health departments should develop region-specific maternal health promotion programs: for example, launching educational campaigns to inform women of childbearing age about the link between MTHFR genotype and folate needs, and distributing genotype-specific supplementation leaflets in community health centers and maternal and child health hospitals. These measures can address the limitations of a “one-size-fits-all” national folic acid supplementation strategy and improve the precision of public health interventions.

Studies have shown that the frequency of the MTHFR C677T polymorphism differs significantly between different regions and ethnic groups around the world. The distribution of the MTHFR C677T polymorphism differs around the world. The frequency of the T allele in Europeans and North Americans is usually higher than Africans and East Asians; there is even a geographical gradient in regions such as Europe, North America, and India (Yang et al., 2017). There have also been many studies investigating the geographical and ethnic distribution of the MTHFR C677T polymorphism in China, but the results are still controversial. The total frequency of the T allele in China is 36.9%, exceeding many other countries (Yang et al., 2017). Further stratified analysis showed that there were significant differences in T allele frequencies among different provinces and ethnic groups. At the same time, as latitude or longitude increases, the frequency of the T allele in China first increases and then decreases. These geographic gradients have previously been observed in other regions such as Pakistan, India, Europe, North America, and East Asia. Several hypotheses have been proposed to explain this difference in distribution, and it is now generally accepted that the distribution pattern of this polymorphism may be caused by natural selection of genetic background and environmental factors (especially folate intake and ultraviolet radiation) (Wang, et al., 2012). In the Chinese population, the total overall frequencies of MTHFR 677 TT genotypes and alleles were 15.0% and 36.9%, respectively (Table1). Studies have confirmed that the distribution of polymorphisms varies across different provinces in China and exhibits obvious geographical trends (Yang et al., 2017): As the latitude increases, the frequency of TT first increases and then decreases (6.6% in the southern region, 14.2% in the central region, 14.2% in the northern region, and 21.3% in the northernmost region); as longitude increases, the TT frequency first increases and then decreases (for example, the TT frequency in the western region is 11.1%, the central region is 20.5%, and the eastern region is 14.0%). In addition, MTHFR 677 TT genotype and allele frequencies also differ between different ethnic groups (Yang et al., 2017). Our study found that the MTHFR C677T genotype and allelic distribution of Han women of childbearing age in Xi’an, Shaanxi are related to those in Xianyang, Ankang, Shangluo, Hanzhong, Yulin, Baoji, Yan’an, Wuhan, Zhoushan, Baiyin City, Zhuhai, Qionghai, Zhenjiang, Kunming, and Yanbian, Jiujiang, Qiqihar, Changsha, Lingshui County, Renshou County, Yinchuan, Taizhou, Guangzhou, Huizhou, Nanchang, Xiangtan, Liupanshui, Nanning City, Lanzhou, Haikou City, Xinjiang Tacheng, Shanghai, Yunnan Dali, Xiamen, Urumqi, Tibet The differences in Lhasa are all statistically significant, but there is no statistical significance when compared with Tongchuan, Cangzhou, Yantai, Changchun and Chifeng in Inner Mongolia (*P* > 0.05). From the overall distribution of MTHFR C677T genotypes in various regions, TT homozygous mutants show a trend of being higher in the north and lower in the south, and the T allele frequency has a downward trend with the decrease in latitude. This study shows that there are certain differences in metabolotypes between different regions in China, and there are also differences in the phenotypic distribution of MTHFR C677T genes in Han women of childbearing age in different regions. International organizations recommend the standard dose of folic acid for individuals with the TT genotype (Hickey et al., 2013; Undas et al., 2019; Huang et al., 2017). Results from a 2021 prospective intervention study showed that supplementing women with different MTHFR gene mutations with multivitamins containing 0.8 mg of folic acid significantly increased serum folate levels, reduced homocysteine (Hcy) levels, and may help lower the risk of neural tube defects (NTDs) in offspring (Kuroda et al., 2021). Expert consensus in China suggests that for women planning pregnancy who are at high risk of folate deficiency-related birth defects and have MTHFR gene TT or CT genotypes (confirmed by genetic testing), it is recommended to take multivitamins containing 0.8–1 mg of folic acid daily, starting at least 3 months before conception (Chen et al., 2024). Therefore, MTHFR C677T genetic testing provides a scientific basis for personalized and precise folic acid supplementation for women of childbearing age to better protect the health and safety of mothers and infants.

## 5 Conclusion

In summary, the study indicates that among Han women of childbearing age in Shaanxi Province, the MTHFR C677T genotype is predominantly CT, with the T allele being the main allele. There are statistically significant differences in the distribution of MTHFR C677T genotypes and alleles among the 8 regions in Shaanxi. Additionally, this study conducted a comparative analysis of MTHFR C677T gene polymorphism among Han women of childbearing age in Xi’an, Shaanxi, with those in other regions of China. The results indicated that the distribution in Xi’an is distinct from many major Chinese populations, particularly those in the south, and that it shares similarities with certain northern populations. It was also found that the frequency of the TT homozygous mutant genotype shows a trend of being higher in the north and lower in the south, and the frequency of the T allele tends to decrease with the decline in latitude. The results of MTHFR C677T gene detection can be used for screening pre-pregnancy and pregnancy risk factors in Han women of childbearing age in Shaanxi Province, providing a certain reference for scientifically and reasonably formulating precise and personalized folic acid supplementation plans.

## Data Availability

The datasets analyzed during the current study including accession information for the raw genotyping data are available from the corresponding author upon reasonable request.
